# Impact of Non-Native Terrestrial Mammals on the Structure of the Terrestrial Mammal Food Web of Newfoundland, Canada

**DOI:** 10.1371/journal.pone.0106264

**Published:** 2014-08-29

**Authors:** Justin S. Strong, Shawn J. Leroux

**Affiliations:** 1 Department of Biology, Memorial University of Newfoundland, St. John’s, NL, Canada; 2 Biology Department, Concordia University, Montreal, QC, Canada; U.S. Geological Survey, United States of America

## Abstract

The island of Newfoundland is unique because it has as many non-native terrestrial mammals as native ones. The impacts of non-native species on native flora and fauna can be profound and invasive species have been identified as one of the primary drivers of species extinction. Few studies, however, have investigated the effects of a non-native species assemblage on community and ecosystem properties. We reviewed the literature to build the first terrestrial mammal food web for the island of Newfoundland and then used network analyses to investigate how the timing of introductions and trophic position of non-native species has affected the structure of the terrestrial mammal food web in Newfoundland. The first non-native mammals (house mouse and brown rat) became established in Newfoundland with human settlement in the late 15^th^ and early 16^th^ centuries. Coyotes and southern red-backed voles are the most recent mammals to establish themselves on the island in 1985 and 1998, respectively. The fraction of intermediate species increased with the addition of non-native mammals over time whereas the fraction of basal and top species declined over time. This increase in intermediate species mediated by non-native species arrivals led to an overall increase in the terrestrial mammal food web connectance and generality (i.e. mean number of prey per predator). This diverse prey base and sources of carrion may have facilitated the natural establishment of coyotes on the island. Also, there is some evidence that the introduction of non-native prey species such as the southern red-backed vole has contributed to the recovery of the threatened American marten. Long-term monitoring of the food web is required to understand and predict the impacts of the diverse novel interactions that are developing in the terrestrial mammal food web of Newfoundland.

## Introduction

Non-native species invasions are a major threat to the persistence of native biodiversity and ecosystem functioning [1]–[3]. The problem is pervasive as invasive species from most major taxonomic groups including plants (e.g. [4]), invertebrates (e.g. [5]), amphibians (e.g. [6]) and mammals (e.g. [7]) have established themselves in many of the World’s biomes. The rate of biological invasions has increased with our increasing human footprint and in some regions the number of non-native species outnumbers the number of native ones. The majority of studies of biological invasions focus on how invasive species and recipient ecosystem traits influence the dispersal and establishment of one or a few non-native species [8], [9]. Few studies, however have investigated the impacts of a community of non-native species on native populations and even fewer have addressed community and ecosystem properties [10], [11]. Consequently, there is a pressing need to understand and predict the impacts of non-native species assemblages on native community and ecosystem properties.

**Figure 1:**
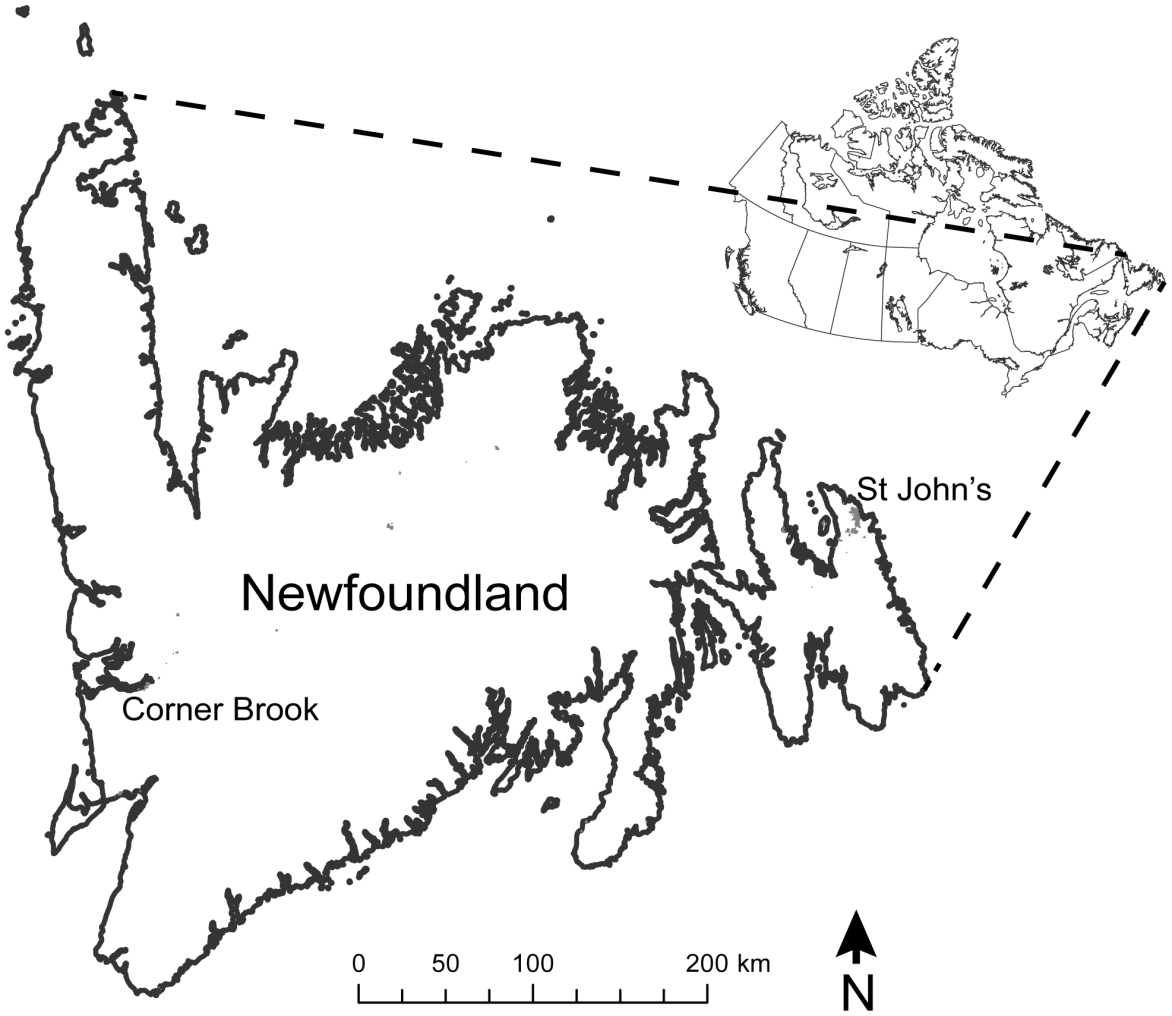
Island of Newfoundland. Map of the island of Newfoundland with a map of Canada inset.

The island of Newfoundland, Canada (Fig. 1) presents a case where the number of non-native and transient terrestrial mammals outnumbers the number of extant native terrestrial mammals. Specifically, there are 13 extant terrestrial mammal species native to the island compared to 13 non-native and 3 transient mammals to the island (Table 1). Native mammals include American Beaver (*Castor canadensis*), American Black Bear (*Ursus americanus*) and American Marten (*Martes americana*). The Grey Wolf (*Canis lupus*) is native to Newfoundland but was extirpated in the early 1930s [12]. Among the introduced non-native mammals are the particularly abundant Red Squirrel (*Tamiasciurus hudsonicus* – introduced in 1963), Snowshoe Hare (*Lepus americanus* – introduced in 1864), and American Moose (*Alces americanus* – introduced in 1904). The Coyote (*Canis latrans*), arrived in 1985 after crossing on ice from Nova Scotia and has established itself on the island through natural range expansion (Table 1). These invasive species may have a large role in the terrestrial mammal food web, where species such as moose may now be found at considerably high densities [13], [14]. In this study, we investigated the impact of the non-native mammal assemblage on the structural properties (e.g. link density, connectance) of the terrestrial mammal food web in Newfoundland.

**Table 1:** Extant native terrestrial mammals on the island of Newfoundland with sources for dietary data used in our study.

Common name	Scientific name	Order	Data originα
American Beaver F2	*Castor canadensis*	Rodentia	Newfoundland
American Black Bear F3	*Ursus americanus*	Carnivora	Newfoundland
American Marten F3	*Martes americana*	Carnivora	Newfoundland
Arctic HareF1	*Lepus arcticus*	Lagomorpha	Newfoundland
Canadian Lynx F3	*Lynx canadensis*	Carnivora	Newfoundland
Caribou F2	*Rangifer tarandus*	Artiodactyla	Newfoundland
Common Muskrat F2	*Ondatra zibethicus*	Rodentia	Maine, New York
Ermine F3	*Mustela erminea*	Carnivora	Newfoundland
Little Brown Myotis F1	*Myotis lucifugus*	Chiroptera	Illinois
Meadow Vole F2	*Microtus pennsylvanicus*	Rodentia	Newfoundland
North American River Otter F3	*Lontra canadensis*	Carnivora	Newfoundland
Northern Myotis F1	*Myotis septentrionalis*	Chiroptera	Illinois, Central Appalachians
Red Fox F3	*Vulpes vulpes*	Carnivora	Newfoundland

α see [53] for full details on data.

F1 Herbivores and insectivores without predators (*F_1_* functional group).

F2 Herbivores and insectivores with predators (*F_2_* functional group).

F3 Predators (*F_3_* functional group).

Common and scientific species names follow Wilson and Reeder [83] except for Caribou.

Compared with mainland regions, islands can be more susceptible to disturbances from the arrival of exotic species [15]. This is due in part to fewer species, lack of natural parasites or predators, more endemic species, and simpler food webs on islands [16]. These traits have generally led to higher rates of species extinctions in insular ecosystems [16], [17]. Biological invasion is advanced as one of the leading causes of biodiversity loss and disturbance on island ecosystems [18]. Specifically, the introduction of non-native mammals can have significant impacts on the flora and fauna of insular ecosystems [19], [20]. For instance, the introduction of the Small Asian Mongoose (*Urva auropunctata*) to Mauritius, Fiji, West Indies, Caribbean islands, and Hawai’i has been catastrophic for some species endemic to these islands. Originally introduced to control rat populations, this non-native mammal has preyed heavily on native fauna [21]. In the Caribbean islands alone, the decline or extinction of 14 of 16 skink species can be attributed to the Small Asian Mongoose [22]. Multiple evolutionary explanations exist for why insular species are sensitive to disturbances from alien species. For example, insular species may be maladapted for competing and co-existing with non-native species; evolution in geographic isolation may result in a lack of traits that facilitate persistence when subject to predation and interference pressures from non-native species [23]. Islands may also support an abundance of resources to allow the spread of non-native species. Therefore, non-native species may rapidly become established and proliferate in island ecosystems, often reaching abundances that are unsustainable within the environment [16]. Although Newfoundland is a continental island and not a “true” island (i.e. oceanic), there is evidence that biodiversity on continental islands also is susceptible to human disturbances such as species introductions (e.g. Australia) [24], [25].

Previous studies have proposed that the effects of non-native species on native food webs are mediated by trophic position (reviewed in [10]). Non-native predators may be especially detrimental for native biodiversity in some areas [25]. For example, a recent meta-analysis showed that non-native predators have a much larger negative effect on population size and reproduction of vertebrate prey than native predators [25]. Conversely, the introduction of non-native prey may confer positive effects on native predators as they can act as viable food sources (reviewed by [26]).

The order of establishment of sequential species introductions also may influence how a non-native species assemblage affects a native food web [27], [28]. For example, Pope et al. [27] show that salmonids introduced to the Klamath Mountains region of California as early as the 1800s have facilitated the establishment and expansion of the non-native Pacific Coast Aquatic Garter Snake (*Thamnophis atratus*) with negative impacts on native Cascades Frogs (*Rana cascadae*). The idea of synergistic or facilitative interactions between non-native species is central to the concept of an “invasional meltdown” (sensu [29]) and evidence of an invasional meltdown has been demonstrated in systems such as the Great Lakes [30].

Ecological network analysis has been applied widely to assess the impact of species loss on food web structure, stability and function (e.g. [31]–[34]). Several recent studies have applied network analysis to investigate the impact of species or groups of species, particularly parasites, on food web properties such as connectance, link density, and prey:predator ratio [9], [35]–[37]. Woodward and Hildrew [38] applied network analysis to investigate the impacts of an invasive predator on native stream food web properties. However, we are aware of few studies that apply network analysis to investigate the impacts of a community of invasive mammal species on native food web properties (but see examples from insect communities in [39], plant-herbivore communities in [40], and plant-pollinator communities in [41]). Studies of specific introduced species to Newfoundland such as American Moose and Red Squirrel have demonstrated significant impacts of these herbivores on forest regeneration and structure [13], [14]. Our goal is to assess the impact of the full suite of non-native terrestrial mammals on the island’s terrestrial mammal food web. Specifically, our research goals are: 1) assemble the first terrestrial mammal food web for the island of Newfoundland, 2) assess impacts of non-native mammals on native mammal food web properties over time, and 3) determine whether changes to food web structure arising from the addition of non-native species can be attributed to specific trophic positions. We hypothesized that the trophic position (e.g. herbivore, predator) of introduced species is the main determinant of its impact on food web structure.

## Materials and Methods

### Food web construction

We consulted the Government of Newfoundland and Labrador’s Department of Conservation and the Environment’s list of terrestrial mammals of Newfoundland [42] for our list of terrestrial mammals on the island. To create a food web describing feeding relationships among these species we extracted data from published studies that involved diet analyses for mammal species on the island. We performed an online search in Web of Science and the Memorial University of Newfoundland online library catalogue for peer-reviewed journals, using the keywords “diet” or “food habits” in combination with “Newfoundland” and the species in question (e.g. “American Black Bear”, “Red Fox”). For mammal species without diet analyses for Newfoundland, we used diet data from other studies mostly from the Canadian boreal region (Table 1). We included non-mammal prey items in our food web.

The studies we reviewed used various techniques to infer the diet of each species including visual (e.g. [43]) and DNA identification [44] of scat contents, stomach-contents analysis (e.g. [45]) stable-isotope analysis (e.g. [46]), and quantitative fatty-acid-signature analysis (e.g. [47]), as well as various forms of field observation (e.g. evidence of browsing on vegetation [48]). The measures used in those studies include percent frequency of occurrence of various food items within total sample or within a specified subcategory of the total sample (e.g. percent frequency of occurrence within samples containing bird remains, [49]), percent of total volume on a year-round and per-season basis (e.g. total percent volume per season, [45]), mean percent prey composition (e.g. [50]), mean percent relative density (e.g. [51]), percent of dry fecal weight (e.g. [52]), percent contribution to fatty acid signatures (e.g. [47]), as well as measures pertaining to field observations, such as percent of stems utilized in an area and percent composition of browse pile (e.g. [48]). Where available, we collected information on sample size, season, and year. From the resulting database, we created a matrix that identified all feeding links among the terrestrial mammal assemblage in Newfoundland. The detailed diet and feeding links databases are available on figshare [53].

The studies we collected showed considerable variability in the resolution at which food items and groups of food items were presented. For example, Riewe [54] categorized vegetation in Red Fox (*Vulpes vulpes*) diet as grasses, *Abies*, or berries, whereas Bridger [55] simply reported on the single class “vegetation”. We grouped food items into categories deemed appropriate to examine the effects of non-native mammals on native mammal feeding interactions, some groups being at a different resolution than others. Ultimately these groups were defined based on the lowest resolution of each food item for each species. Data on mammals are reported at the species level. Data on bird food items are reported to the level of Order (members of the Orders Anseriformes, Columbiformes, Galliformes, Gruiiformes, Passeriformes), except aquatic birds (a group that contains the Orders Charadriiformes, Procellariiformes, and Pelecaniformes). The category of aquatic birds is the finest resolution to which we could classify these food items.

Plant food items were designated as grasses, herbaceous plants, mosses, lichens, fungi, fruits or deciduous shrubs/trees. Balsam Fir (*Abies balsamea*) was placed in its own category apart from the other evergreen shrubs and trees because it is an important dietary component for many species in Newfoundland [56]–[59]. Our category “herbaceous plants” includes freshwater aquatic plants, “fruits” includes berries, and “mosses” includes mosses and other bryophytes. The food item “terrestrial invertebrates” includes insects and “marine mammals” includes only cetaceans and pinnipeds.

The remaining food item groups were marine invertebrates and fish. Some diet studies used broad or vague categories (e.g. “achlorophyllous plant matter”, “birds”, “bone”, “eggshells”, “feathers”, “flesh”, “garbage”, “green vegetation”, “leaves”, “miscellaneous vegetation”, “nuts/seeds”, “plant material”, “roots”, “seeds”, and “woodchips”). Oftentimes detailed explanations of these groups were not included in the studies. We incorporated those data in our detailed database but not to construct the mammal feeding links matrix. In addition, some studies from outside Newfoundland included diet items that are not present on the island, or are domestic animals (e.g. lemmings, deer, chicken, cow, elk). Again, these data were included in the detailed database only.

### Food web analysis

We calculated species richness (S), total number of links (L), connectance (C = L/S^2^), link density (L/S), fraction of basal species (i.e. species that do not consume other species), fraction of intermediate species (species that consume and are consumed by other species), fraction of top species (i.e. species that are not consumed), prey:predator ratio (i.e. the number of basal and intermediate species divided by the number of predatory and intermediate species), vulnerability (i.e. mean number of predators per prey species; sensu [60]), and generality (i.e. mean number of prey per predator species; sensu [60]). We began by computing these food web properties for the native terrestrial mammal food web only. To assess the influence of non-native species arrivals over time, non-native mammals were added to the native mammals food web sequentially in the order in which they arrived on the island, with non-native species being left in the web as more recently introduced species were added. We then quantified each food web metric for the addition of every new non-native species.

There is considerable empirical evidence relating non-native species functional groups to their ecological impacts (reviewed in [10]). Consequently, we assessed the effect of different non-native functional groups on food web properties by separating native and non-native species into three functional groups defined by our feeding links matrix. The first functional group, *F_1_*, was “Herbivores and insectivores without predators”, the second functional group, *F_2_*, was “Herbivores and insectivores with predators”, and the third functional group, *F_3_*, was “Predators”. Tables 1 and 2 outline which native and non-native mammal species belong to each functional group. We quantified the effect of non-native trophic position on food web properties by first removing each native and non-native mammal from the same trophic group and calculating the following effect size ratios for each metric; (*Full web−F_1,i_*)*/Full web,*; (*Full web−F_2,j_*)*/Full web, and*; (*Full web−F_3,k_*)*/Full web*, where *i*, *j*, *k* are indices representing species from functional groups *F_1_*, *F_2_*, and *F_3_*, respectively. For example, the effect of removing American Moose (functional group *F_2_*) on link density was calculated as link density of the full food web minus link density of the food web without American Moose divided by the link density of the full food web. We calculated the mean effect of removing non-native mammals from each functional group, *x*, on our food web metrics as non-native mean ((*Full web – F_x,i_*)*/Full web*)/native mean((*Full web – F_x,i_*)*/Full web*). This allowed us to tease apart the effect of removing a native vs non-native species from the functional group on food web properties. Wolves were not included in our analyses because most non-native species arrived after the wolf was extirpated from Newfoundland and our focus was to investigate the impacts of non-native species on the native food web. Also, we excluded three transient or vagrant mammals species: Arctic Fox (*Alopex lagopus*), Polar Bear (*Ursus maritimus*), and Hoary Bat (*Lasiurus cinereus*) because they are only seasonal visitors to the island. Finally, we excluded Bank Vole (*Myodes glareolus*) and American Bison (*Bison bison*) from our analyses because they were both introduced to offshore islands with the latter introduction not being successful. All analyses were conducted with the foodweb package [61] in R v 2.15.1 [62]. Gephi [63] was used to construct our food web diagram.

**Table 2:** Non-native and transient terrestrial mammals on the island of Newfoundland with sources for dietary data used in our study.

Common name	Scientific name	Order	Year of introductionφ	Data originα
American Mink F3	*Neovison vison*	Carnivora	1935	Newfoundland
American Moose F2	*Alces americanus*	Artiodactyla	1904	Newfoundland
Arctic Fox	*Alopex lagopus*	Carnivora	transient	Alaska, Greenland, Sweden
Bank Vole	*Myodes glareolus*	Rodentia	1967	excluded
American Bison	*Bison bison*	Artiodactyla	1964	excluded
Cinereus Shrew F2	*Sorex cinereus*	Soricomorpha	1958	Ontario
Coyote* F3	*Canis latrans*	Carnivora	–	Newfoundland
North American Deermouse F1	*Peromyscus maniculatus*	Rodentia	<1968	Ontario, Virginia, Indiana
Eastern Chipmunk F1	*Tamias striatus*	Rodentia	1962	Ohio
Hoary Bat	*Lasiurus cinereus*	Chiroptera	transient	Manitoba
House Mouse F1	*Mus musculus*	Rodentia	with human settlement	Indiana
Brown Rat F2	*Rattus norvegicus*	Rodentia	with human settlement	Alaska
Polar Bear	*Ursus maritimus*	Carnivora	transient	Canadian Arctic
Red Squirrel F2	*Tamiasciurus hudsonicus*	Rodentia	1963	Quebec
Snowshoe Hare F2	*Lepus americanus*	Rodentia	1864	Newfoundland
Southern Red-Backed Vole F2	*Myodes gapperi*	Rodentia	1998	Ontario

*Coyote arrived on the island of Newfoundland through natural range expansion in 1985.

φ From [42], except southern red-backed vole [66].

α See [53] for full details on data.

F1 Herbivores and insectivores without predators (*F_1_* functional group).

F2 Herbivores and insectivores with predators (*F_2_* functional group).

F3 Predators (*F_3_* functional group).

## Results

The extant native terrestrial mammals of Newfoundland come from the orders Carnivora (6 species), Rodentia (3 species), Chiroptera (2 species), Artiodactyles (1 species), and Lagomorpha (1 species). We obtained dietary data on 10 of these species from studies conducted on the island of Newfoundland (Table 1). The non-native terrestrial mammals of Newfoundland come from the orders Rodentia (7 species), Carnivora (4 species), Artiodactyles (1 species), Chiroptera (1 species), and Soricomorpha (1 species). We obtained dietary data for 4 of these non-native species from studies conducted on the island of Newfoundland (Table 1). The remaining non-native mammal diet data came from elsewhere in North America (e.g. Ontario, Alaska). The diet data reveals that the 11 non-native terrestrial mammal species considered in our food web analysis (i.e. excluding the 3 transient species) have, on average, 5 different resource types (sd = 2.19) whereas the native terrestrial mammal species have, on average, 7.08 different resources (sd = 5.65).

**Figure 2:**
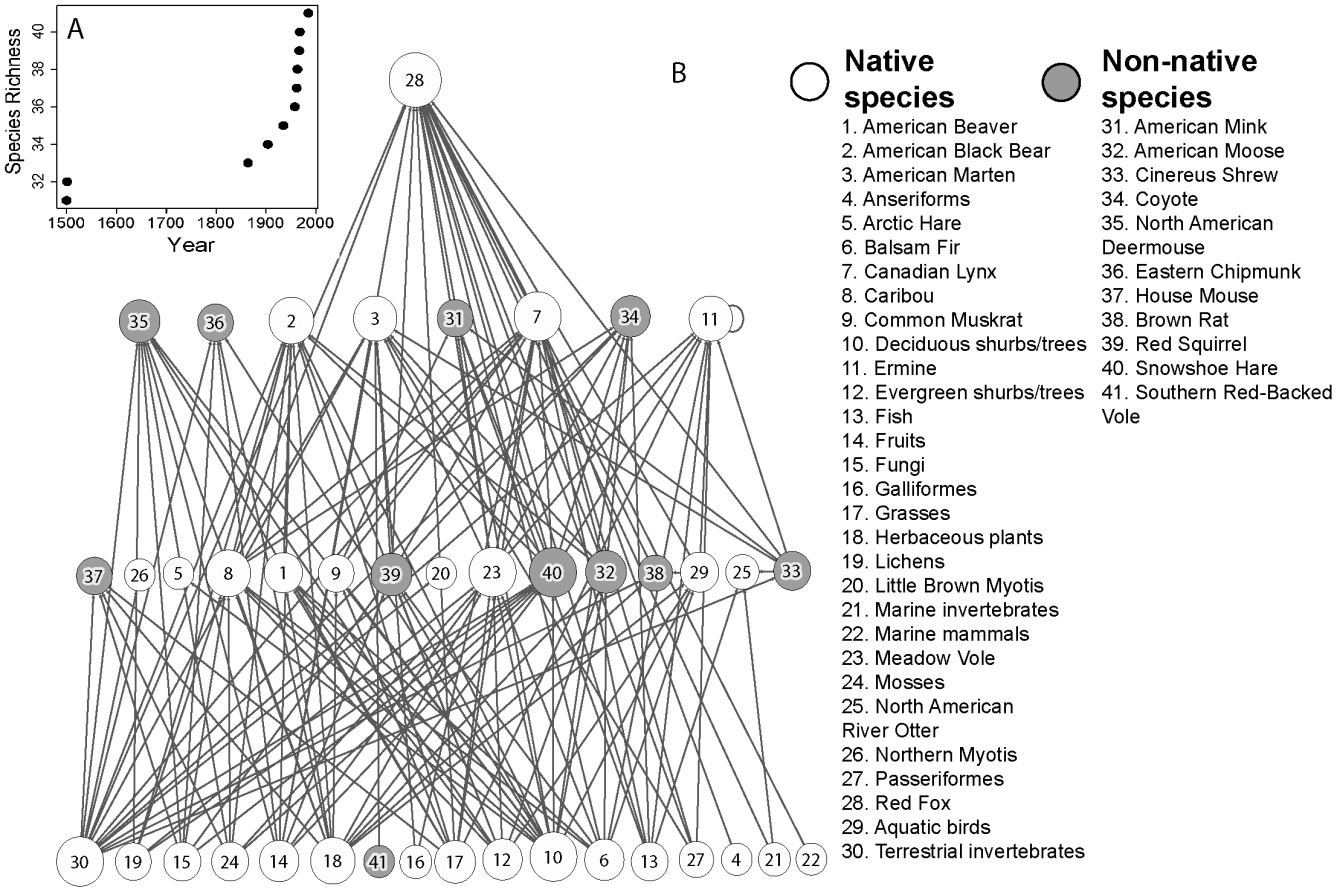
Terrestrial mammal food web of Newfoundland. A, year that each non-native species considered in our study was introduced vs total terrestrial mammal species richness in Newfoundland. B, Newfoundland terrestrial mammal food web with native and non-native terrestrial mammals shaded in different colours. Each node represents a different species or group of species and each link represents a feeding relationship. For example, terrestrial invertebrates (30) are prey for Cinereus Shrew (33).

**Figure 3:**
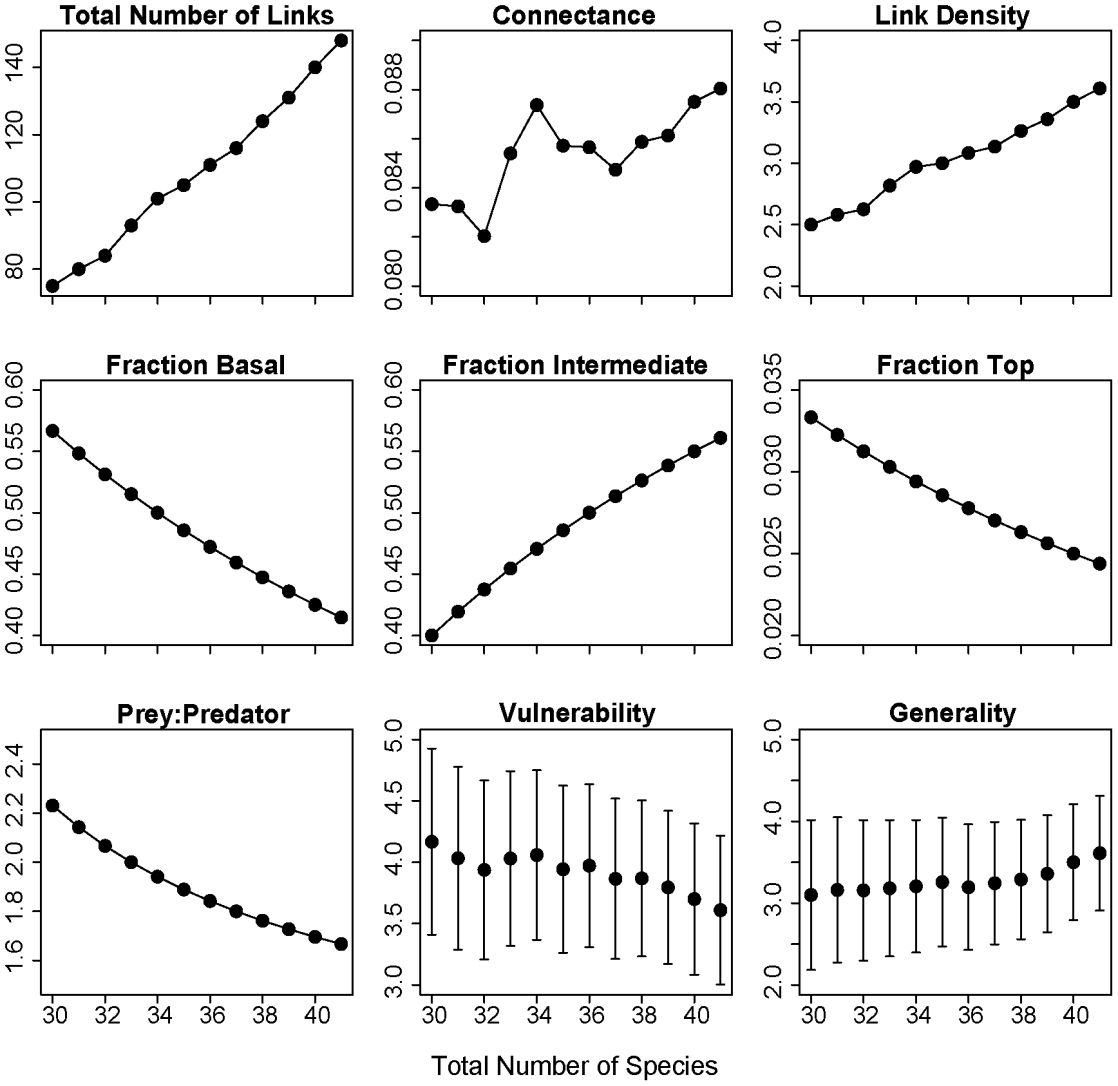
Effects of non-native mammals on food web properties. Change in terrestrial mammal food web properties with the sequential addition of non-native species on the island of Newfoundland. The native food web has 30 species and every point after this represents the addition of one non-native species added in chronological order (see Table 2).

The earliest non-native mammal species to colonize Newfoundland (Fig. 1) were the House Mouse and Brown Rat which arrived with human settlement in the late 15^th^ and early 16^th^ centuries (Table 2). The Southern Red-Backed Vole is the last non-native mammal species to colonize the island ca. 1998 (Table 2). There are 30 species (including basal resource categories) in the native mammal food web of Newfoundland and 41 species after the establishment of 11 non-native mammals on the island (Fig. 2). As expected, the total number of links and link density in the food web increased steadily with the addition of non-native mammals over time (Fig. 3). The trend in connectance over time also was positive but this trajectory was variable with the addition of some non-native mammals leading to a decline in connectance. The fraction of intermediate species in the food web increased from 0.4 to 0.56 with the addition of non-native mammals over time (Fig. 3). As more intermediate species colonized the island, the fraction of basal and top species in the food web declined from 0.57 to 0.41 and 0.03 to 0.02, respectively. The prey:predator ratio in the food web declined steadily from 2.23 to 1.67 with the addition of non-native mammals to the food web. Finally, food web vulnerability declined from 4.17 to 3.61 and generality increased from 3.1 to 3.61 with the arrival of non-native mammals to the island (Fig. 3).

**Figure 4:**
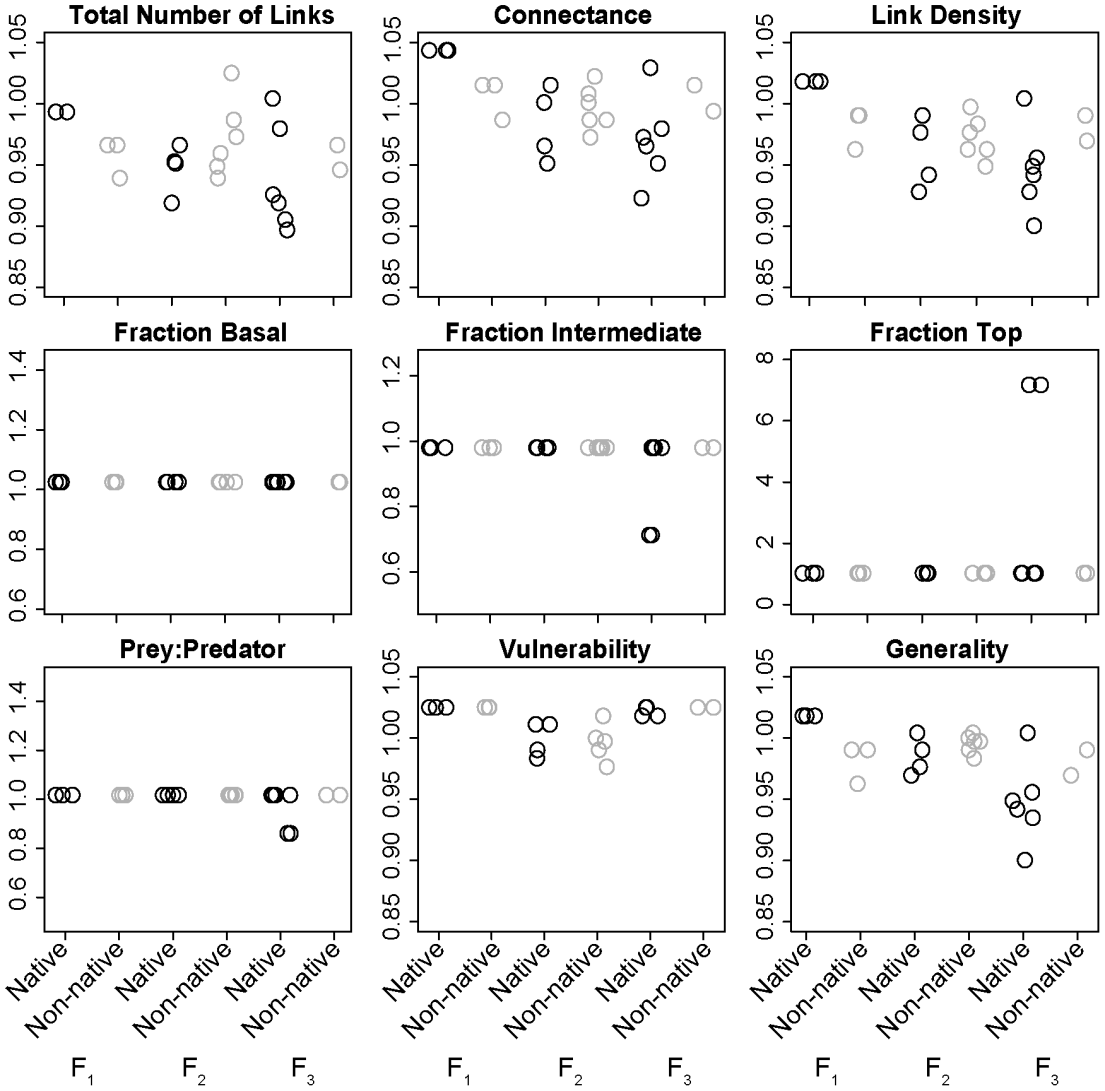
Effects of non-native mammal functional groups on food web properties. Change in Newfoundland terrestrial mammal food web properties for the removal of native vs non-native species of herbivores and insectivores without predators (*F_1_*), herbivores and insectivores with predators (*F_2_*), and predators (*F_3_*). See methods for specific definitions of *F_1_*, *F_2_*, and *F_3_*.

The removal of non-native herbivores and insectivores without predators (i.e. *F_1_*) led to lower (0.96 x) total number of links, connectance, link density and generality than the removal of native mammals from the same functional group (Fig. 4). There was no difference in the fraction of basal, fraction of intermediate, fraction of top, prey:predator ratio, and vulnerability to the loss of non-native vs native herbivores and insectivores with or without predators (Fig. 4). The removal of non-native herbivores and insectivores with predators led to a small increase (1.01–1.02 x) in the total number of links, connectance, link density, and generality than the removal of native mammals from the same functional group (Fig. 4). The removal of non-native predators led to an increase (1.02–1.05 x) in the total number of links, connectance, link density, fraction of intermediate, prey:predator ratio, and generality, a decrease in the fraction of top (0.33 x) and no change in the fraction of basal and vulnerability compared to the removal of native predators (Fig. 4).

## Discussion

The island of Newfoundland has as many non-native terrestrial mammals as native terrestrial mammals (Table 1, 2). A few studies have investigated the impacts of non-native American Moose, Red Squirrel and Snowshoe Hare on the island’s flora (e.g. [13], [14]) but no studies have investigated the impact of the full assemblage of non-native terrestrial mammals on Newfoundland’s mammal food web structure. Our diet and network analysis of terrestrial mammals in Newfoundland confirmed our hypothesis that the trophic position of non-native species would be the main determinant of non-native species impacts on food web structure. Specifically, our data show that non-native mammals have influenced the island’s terrestrial mammal food web primarily by increasing the fraction of intermediate (i.e. herbivores and insectivores) species, which has led to increasing food web connectance and generality (Fig. 3).

The colonization of 11 non-native mammals to Newfoundland has resulted in a terrestrial mammal food web with 1.3x the number of carnivore species, 2x the number of herbivore and insectivore species without predators and 3x the number of herbivore and insectivore species with predators compared to the native mammal food web (Fig. 2, 3). These changes to food web structure has led to greater connectance in the terrestrial mammal food web on the island because, on average, herbivores and insectivores have more food web links than predators. Recent theoretical work based on the analysis of Jacobian matrices suggests that an increasing number of prey species per predator may confer stability to food webs [64]. In Newfoundland, non-native species introductions have led to a threefold increase in the number of prey species per predator species and there is already evidence that a greater prey base has potentially contributed to the recovery of a native top predator species on the island. Specifically, American Marten were listed as endangered by the Committee on the Status of Endangered Wildlife in Canada (COSEWIC) in 1996 but a re-assessment in 2007 resulted in marten being listed in a lower risk category: threatened [56]. Introduced Snowshoe Hares and Southern Red-Backed Voles may be leading to a more diversified diet for marten and contributing to its recovery [65], [66]. However, other factors such as reduced mortality due to a decline in snaring by-catch and habitat loss also are contributing to marten recovery [56].

While the introduction of prey species may confer some benefits to the terrestrial mammal food web in Newfoundland, it is too early to determine the long-term effects of these introductions on the native mammal assemblage. For example, abundant non-native moose may provide ample carrion to support Coyotes and this has the potential to increase predation pressure on the native Caribou population. This apparent competition between moose and caribou via canid predators has been observed in other boreal ecosystems [67], [68].

The Coyote is one of the most recent species to establish itself in Newfoundland. In most of their range, Coyotes have a diverse diet consisting of a number of small- to medium-sized mammals (e.g. snowshoe hare, voles) and carrion (e.g. [69], [70]). We suggest that the large increase in terrestrial mammal prey and carrion base in Newfoundland may have facilitated the successful establishment of Coyotes to the island. If this interpretation is true, we may have the first evidence of an invasional meltdown (sensu [29]) in Newfoundland where positive interactions among non-native species could lead to cumulative impacts on native ecosystems and the facilitation of additional non-native species colonizations. The indirect effects of Coyote predation on native mammals in Newfoundland should be a focus of future terrestrial mammal studies because this mesopredator has significant impacts on food webs in other ecosystems [71], [72].

It may seem trivial that prey must be present on an island or patch in order for predators to successfully establish themselves (naturally or via human introduction). But ecologists have built this fundamental trophic interaction constraint into theoretical models only recently [73], [74]. For example, in the trophic theory of island biogeography, predators can only colonize and persist on islands that are already occupied by their main prey species [73]. The inclusion of this trophic constraint led to improved predictions of real food web community composition compared to the classic theory of island biogeography [73]. Extensions of this theory to consider multiple preys may help explain the persistence of predator populations over large spatial extents.

The full terrestrial mammal food web of Newfoundland with 11 non-native mammals has 1.37x the number of mammals and 1.97x the number of links of the native terrestrial mammal food web of Newfoundland (Fig. 2, 3). However, the removal of non-native herbivores and insectivores with predators or non-native predators led to an increase in the total number of links, connectance, link density and generality compared to the removal of native mammals from these trophic groups (Fig. 4). This suggests that native mammals from these groups are more connected to other species in the food web than non-native mammals. For example, the mean number of prey per native predator (American Black Bear, American Marten, Canadian Lynx, Ermine, North American River Otter, Red Fox) is 11.2 whereas the mean number of prey per non-native predator (Coyote, American Mink) is 6.5. Conversely, the removal of non-native herbivores and insectivores without predators led to a decline in the total number of links, connectance, link density and generality compared to the removal of native species from this trophic group (Fig. 4). This suggests that non-native herbivores and insectivores without predators were more connected to other species in the food web than native mammals from this trophic group. Indeed, all three native herbivores and insectivores without predators (Arctic Hare, Little Brown Myotis, Northern Myotis) only have one prey item each whereas the three non-native herbivores and insectivores without predators (Eastern Chipmunk, North American Deermouse, House Mouse) have on average 6.33 prey items each.

Connectance and link density of species in food webs are primary determinants of the robustness of food webs to species losses or additions [31], [34], [36], [64], [75]. Species that have many links to other species in a food web have a greater potential to influence community structure [31]. But recent research highlights that the connectivity, trophic level, and traits of species removed (or added) will determine the cascading impacts of removing (or adding) this species [31], [36], [64]. For example, the loss of a prey species in a web with generalist predators and a diverse prey base may lead to few secondary extinctions [76]. Our study differs from most other food web network analyses in that we are concerned with the impacts of species addition instead of species loss on food web structure. The additions of species will invariably lead to novel trophic interactions [77]–[79] and novel interactions may lead to complex effects on food web properties. For example, the introduction of the Southern Red-Backed Vole may lead to direct competition for resources and space with the native Meadow Vole but also indirect competition (i.e. apparent competition) among these small mammals (sensu [80]) via shared predators (e.g. American Marten). Teasing apart the relative strength of direct and indirect competition among species may be key for understanding species coexistence [81]. Long-term monitoring of populations is required to understand the impacts of novel interactions in the terrestrial mammal food web of Newfoundland.

This research represents one of the few studies to apply network analysis to investigate the impacts of non-native species on native food web properties. We began by assembling the first terrestrial mammal food web for the island of Newfoundland based on a review of empirical diet analyses studies. Although likely an incomplete representation of the diet of all mammal species in Newfoundland (e.g. we did not include non-mammal predators such as *Falconiformes spp.*), our data represent the best knowledge of the diet of Newfoundland mammals and forms a good starting point for identifying key gaps in diet data. Non-native mammals have resulted in a 2.5x increase in the number of herbivorous and insectivorous terrestrial mammals in Newfoundland. Through network analysis we show that this increase in intermediate species has led to the development of a terrestrial mammal food web with higher connectance and generality. Future work should consider the cumulative effects of global changes such as land-use and climate change and species harvesting along with the arrivals of non-native species on the structure and functioning of the terrestrial mammal food web of Newfoundland [10], [42], [82].
